# HyCHEED System for Maintaining Stable Temperature Control during Preclinical Irreversible Electroporation Experiments at Clinically Relevant Temperature and Pulse Settings

**DOI:** 10.3390/s20216227

**Published:** 2020-10-31

**Authors:** Pierre Agnass, Hans M. Rodermond, Remko Zweije, Jan Sijbrands, Jantien A. Vogel, Krijn P. van Lienden, Thomas M. van Gulik, Eran van Veldhuisen, Nicolaas A. P. Franken, Arlene L. Oei, H. Petra Kok, Marc G. Besselink, Johannes Crezee

**Affiliations:** 1 Department of Radiation Oncology, Cancer Center Amsterdam, Amsterdam UMC, University of Amsterdam, 1105 AZ Amsterdam, The Netherlands; p.agnass@amsterdamumc.nl (P.A.); h.rodermond@amsterdamumc.nl (H.M.R.); r.zweije@amsterdamumc.nl (R.Z.); j.sijbrands@amsterdamumc.nl (J.S.); n.a.franken@amsterdamumc.nl (N.A.P.F.); a.l.oei@amsterdamumc.nl (A.L.O.); h.p.kok@amsterdamumc.nl (H.P.K.); 2 Department of Surgery, Cancer Center Amsterdam, Amsterdam UMC, University of Amsterdam, 1105 AZ Amsterdam, The Netherlands; t.m.vangulik@amsterdamumc.nl (T.M.v.G.); e.vanveldhuisen@amsterdamumc.nl (E.v.V.); m.g.besselink@amsterdamumc.nl (M.G.B.); 3 Laboratory of Experimental Oncology and Radiobiology, Cancer Center Amsterdam, Amsterdam UMC, University of Amsterdam, 1105 AZ Amsterdam, The Netherlands; 4 Center for Experimental Molecular Medicine, Cancer Center Amsterdam, Amsterdam UMC, University of Amsterdam, 1105 AZ Amsterdam, The Netherlands; 5 Department of Gastroenterology & Hepatology, Amsterdam Gastroenterology and Metabolism, Amsterdam UMC, University of Amsterdam, 1105 AZ Amsterdam, The Netherlands; j.a.vogel@amsterdamumc.nl; 6 Department of Radiology, Cancer Center Amsterdam, Amsterdam UMC, University of Amsterdam, 1105 AZ Amsterdam, The Netherlands; k.p.vanlienden@amsterdamumc.nl

**Keywords:** hydraulic-control-system, heating-system, cooling-system, electroporation, electromagnetics, hyperthermia

## Abstract

Electric permeabilization of cell membranes is the main mechanism of irreversible electroporation (IRE), an ablation technique for treatment of unresectable cancers, but the pulses also induce a significant temperature increase in the treated volume. To investigate the therapeutically thermal contribution, a preclinical setup is required to apply IRE at desired temperatures while maintaining stable temperatures. This study’s aim was to develop and test an electroporation device capable of maintaining a pre-specified stable and spatially homogeneous temperatures and electric field in a tumor cell suspension for several clinical-IRE-settings. A hydraulically controllable heat exchange electroporation device (HyCHEED) was developed and validated at 37 °C and 46 °C. Through plate electrodes, HyCHEED achieved both a homogeneous electric field and homogenous-stable temperatures; IRE heat was removed through hydraulic cooling. IRE was applied to 300 μL of pancreatic carcinoma cell suspension (Mia PaCa-2), after which cell viability and specific conductivity were determined. HyCHEED maintained stable temperatures within ±1.5 °C with respect to the target temperature for multiple IRE-settings at the selected temperature levels. An increase of cell death and specific conductivity, including post-treatment, was found to depend on electric-field strength and temperature. HyCHEED is capable of maintaining stable temperatures during IRE-experiments. This provides an excellent basis to assess the contribution of thermal effects to IRE and other bio-electromagnetic techniques.

## 1. Introduction

Irreversible electroporation (IRE) is a relatively new focal ablation technique for the treatment of unresectable solid tumors at challenging anatomical sites including the liver, kidney, and pancreas [1,2,3]. IRE is a modality in which short but high-voltage pulses are applied between electrodes implanted in the target volume to cause nanopores in the cell membranes. The pore formation induces disturbances in the cell’s homeostasis, with cell death as consequence through accidental and regulated cell death mechanisms, and causes an increase of the electrical specific conductivity in the bulk tissue due to improved solute mobility [4,5].

Contrary to thermal ablation modalities, such as radiofrequency ablation, microwave ablation, and cryoablation, IRE is assumed to have the advantage of being less prone to local cooling by the heat-sink effect [6]; an effect that is known to hamper the successful treatment of thermal ablation modalities due to underdosage near large blood vessels in the ablation zone [7]. In addition, IRE preserves the vital structures (e.g., blood vessels, bile ducts) traversing the ablation zones, since fibrous and collagen structures are typically not affected by IRE [6].

Despite the fact that IRE was initially introduced as a non-thermal technique, the application of repetitive high-intensity electrical pulses in the target organs, inevitably leads to increased temperatures during ablation [8,9,10,11]. The produced thermal effects could have the potential to enhance the effectiveness of IRE treatment, since mild hyperthermia and thermal ablation are known to have therapeutic effects. Specifically, mild hyperthermic temperatures (T ≤ 45 °C) enhance the therapeutic effects of chemotherapy and radiotherapy, depending on the temperature and the duration of heating, i.e., the thermal dose [3,12,13,14,15,16]. These enhancements are achieved through a variety of molecular and physiological mechanisms, including inhibition of DNA damage repair for T > ~41 °C, increase of blood flow and reoxygenation for 39 °C < T < 44 °C, decrease of tumor pH, and stimulation of the immune response [15,17,18,19]. Direct cell kill will occur at ablative temperatures, e.g., for short duration exposure to T ≥ 50 °C [8,20,21]. The temperature increase during traditional mild hyperthermia and thermal ablation is produced using invasive or external techniques, such as electromagnetic, capacitive, conductive heating, or focused ultrasound techniques [3,22,23,24,25,26].

During IRE, the produced thermal effects at mild hyperthermic temperature levels have the potential to enhance the irreversible permeabilization effects (IPE) by reducing the resistance of the tissue and the electric-field threshold for nanopore formation (E_IRE(th)_) [27]. We chose IPE instead of IRE effect to distinguish between only the permeabilization effect, and the permeabilization and the thermal effects jointly produced by IRE. For higher temperature levels (T ≥ 50 °C [8,20,21]), even direct thermal damage could occur, which can result in thermal ablation, especially in the vicinity of the needle electrodes [8,9,10,11]. However, it remains unclear to what extent the thermal effects of IRE contribute to tissue ablation. A better understanding of this contribution can help to establish IRE settings that maximize the ablative tumor effect.

Establishing the contribution of thermal effects requires performing pre-clinical IRE experiments while maintaining a well-defined, stable temperature. The latter is challenging, as the very process of IRE can cause an unpredictable temperature rise [8,9,10,11,20], which needs to be dealt with to achieve stable experimental conditions. Several studies developed setups that aimed at maintaining the temperature of the target volume at a specific target temperature (T_target_ [°C]) during IRE [27,28]. For example, Edelblute et al. attempted to heat and maintain the temperature of their target volumes at a desired temperature using a heat block or time-modulated fiber optic laser [27]. In the case of Zhang et al., the desired temperatures were achieved by preheating the target using a radiofrequency ablation technique [28]. Overall, temperature maintenance in the target volume was achieved due to the controlled heat exchange between the target volume and ambient environment that resulted in temperature fluctuations of 2–5 °C with respect to the T_target_. However, the needle electrodes generally utilized in IRE studies inevitably yield spatially heterogeneous electric-field and temperature distributions, with higher energy deposition close to the needles. To thoroughly investigate the influence and dose–effect relationships of both the electric-field and thermal distribution, it is important to maintain the electrical and thermal parameter levels spatially as homogeneously as possible for all the various temperature and pulse settings used, while maintaining the temperature variation as small as possible. This requires careful balancing of the heat exchange between the target volume and the ambient environment for various pulse settings. This is challenging, since the temperature increase not only varies as function of the pulse settings, but also as function of the T_target_ due to the increase in the electrical and thermal conductivity of the target tissue as a function of both pulse settings and T_target_ [20].

Therefore, the main objective of this paper is to present, demonstrate, and validate a novel temperature-controlled electroporation device capable of maintaining a pre-specified stable temperature level in a target volume (TV) with small temperature variation while performing IRE for several different fixed clinically relevant IRE settings in vitro in between plate electrodes. The second objective is to illustrate how this device can be used to determine the influence of IPE and thermal effects on (1) the cell viability in vitro and (2) the electrical specific conductivity (σ [S·m^−1^]) during and after IRE treatment.

## 2. Materials and Methods

**Figure 1:**
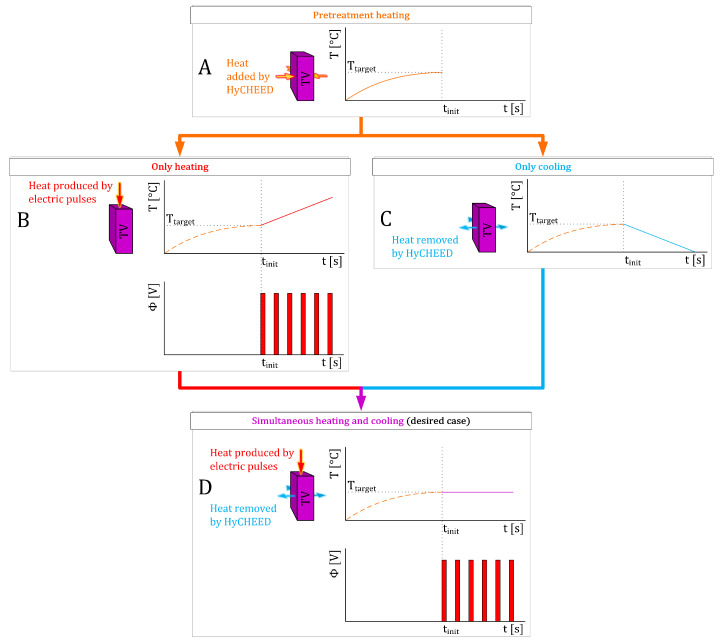
Schematic illustration of the temperature control mechanism of the hydraulically controllable heat exchange electroporation device (HyCHEED). (**A**) The left side illustrates a target volume (TV) represented by a purple cuboid that is heated up by HyCHEED up to the target temperature (T_target_ [°C]) prior to the initial time (t_init_ [s]) at which IRE starts; the heat is represented by orange arrows. The right side shows the temperature (T [°C]) of the TV as a function of time (t [s]). Please note that the black dotted lines represent T_target_ and t_init_. After achieving T_target_ for (**B**–**D**), three situations were presented: Situation 1 (**B**) illustrates the TV in which the heat is generated at t_init_ due to a series of electric pulses with voltage (Φ [V]) in the TV when neglecting any cooling effects; the produced heat due to the electric pulses is represented by a red arrow. Situation 2 (**C**) illustrates the TV from which heat is removed by HyCHEED when neglecting heating by the electric pulses; the heat removal is represented by blue arrows. Situation 3 (**D**; the desired case) illustrates the TV in which a balance is achieved between heat generation due to electric pulses and heat removal by HyCHEED.

A hydraulically controllable heat exchange electroporation device (HyCHEED) was developed to maintain a stable temperature level in a target volume (TV) during electroporation by removing the heat generated by the electric pulses. The main challenge is thus to create a balance between heat generation due to electric pulses and heat removal by HyCHEED. The sophisticated control mechanism developed for this purpose is schematically illustrated in Figure 1 and will be further explained in the next paragraphs. Specifically, the mechanical structure and the mathematical concept of HyCHEED will be explained in Section 2.1. Materials and Instruments and Appendix A—Mathematical Description of Stable Temperature Control in Treated Volume.

### 2.1. Materials and Instruments

HyCHEED consists of (1) a hydraulic unit through which demineralized water (demi-water) flows for controlled heating/cooling of the TV, (2) an electrical unit for application of IRE to the TV, and (3) a control unit that controls the heat exchange in the TV during IRE to achieve and maintain the pre-specified temperature level. In this paper a TV of ~300 μL of cell suspension was treated.

#### 2.1.1. Hydraulic Unit

**Figure 2:**
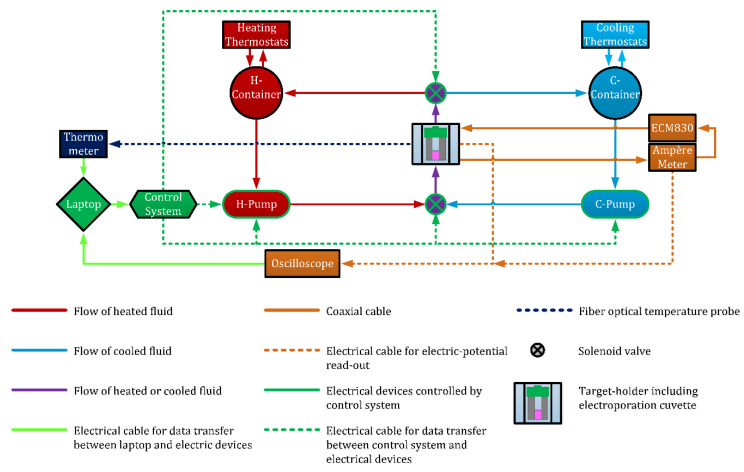
Overview of the controlled hydraulic circuit. The colors red, light blue, and purple represent different parts of the hydraulic unit, the color light brown represents the electric unit, the colors green and light green represent the control unit, and the color dark blue represents fiber optical thermometer. Please note that parts of the hydraulic unit with green lines are controlled by the control unit. Also note that the letters H and C are abbreviations of the words heating and cooling.

The hydraulic circuit pumped demi-water through temperature-controlled containers to the target holder (T-holder; hyperlink to see Section 2.2.). Two water containers were used to control the demi-water temperature to create stable temperature control in the TV under dynamic conditions during IRE—one container at high temperature (H-container) for increasing the temperature in TV and the other at temperature lower than T_target_ (C-container) for achieving cooling when needed. Both containers were filled with demi-water that heated up to appropriate temperature levels, depending on T_target_. For an example, see Table 1. Two peristaltic pumps (VPER-HF, FWT SYSTEMS SRLS, Rome, Italy) placed before the T-holder were used to circulate the demi-water at a stable flow rate inside the T-holder to control the temperature of the TV. See the red, light blue, and purple lines and shapes in Figure 2.

#### 2.1.2. Electric Unit

The cell suspension was treated using an electroporation cuvette (WB 1-4120, Westburg, Leusden, The Netherlands). The cuvette includes two plate electrodes made of aluminum with an inter-electrode distance of 2 mm between which the cell suspension is included. Specifically, the cuvette was placed inside the T-holder to control the temperature of the cell suspension. In the T-holder, the electrodes of the cuvette contact thin copper tapes that are connected to the pulsing circuit. More details about the architecture of the T-holder including the electrode connections are described in hyperlink to Section 2.2. The electric pulsing circuit consists of the pulse generator ECM^®^ 830 (BTX-Harvard Apparatus, Holliston, MA, USA) in series with an electrical resistor of 0.1 Ω for the measurement of electric current and PicoScope3206 (Pico Technology, Saint Neots, UK) to record the electric voltages across the cuvette and the electrical resistor of 0.1 Ω. See the light brown blocks and lines in Figure 2.

#### 2.1.3. Control Unit

Two solenoids valves (Type 0124, Christian Bürkert GmbH & Co. KG, Ingelfingen, Germany) were used to regulate and mix the flow of the heated and the cooled demi-water. A control system was built to control the water flow and valve settings and thereby the temperature of the cell suspension by switching between the solenoids valves and the pumps. In Figure 2, the control unit is indicated as (light) green lines and shapes.

### 2.2. Target-Holder Architecture

**Figure 3:**
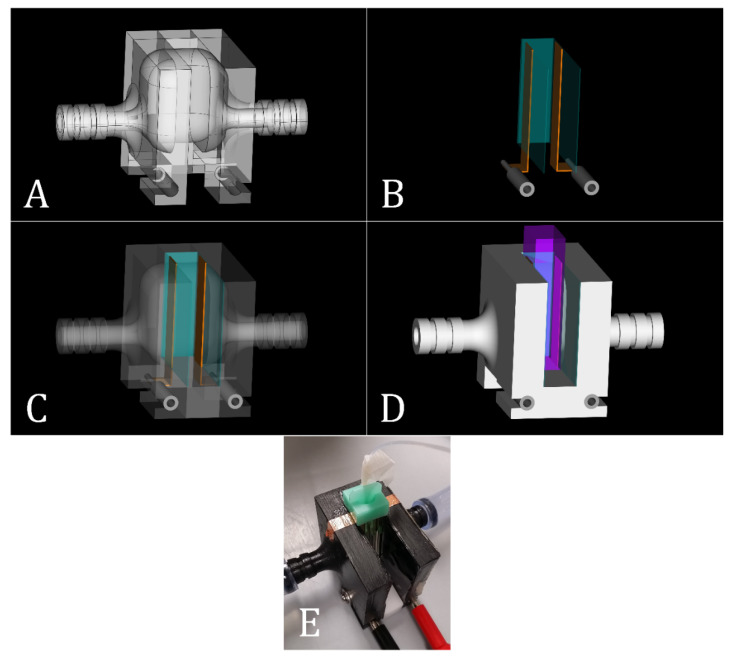
(**A**) A transparent version of the target holder (T-holder) with rounded edges and corners at the inside for optimal water flow. (**B**) Female banana connectors (color gray) connected to copper-film tapes (color brown; with width of 10 mm) that were taped to the water bolus (PVC film; color turquoise). (**C**) Transparent T-holder combined with the electrodes and the water bolus from (**B**). In between the banana connectors, a PLA block was used as a stand support for the electroporation cuvette. (**D**) A complete T-holder model including an electroporation cuvette (color purple). Here, the cuvette can be slid from above or the front along the water bolus and the copper tapes. The electroporation cuvette includes two plate-electrodes with a 2 mm gap that is filled with cell suspension (cells in culture medium). These electrodes contact the copper tapes that are connected to the pulsing circuit for the application of IRE. (**E**) T-holder used during the experiments. The optical probe was inserted inside the catheter, with open ends, placed in the cap of the cuvette. To avoid probe motions, the catheter was pasted on the probe on one end using adhesive tape, after which the tape was disinfected.

The T-holder architecture consists of (1) a small container and a water bolus to obtain and maintain T_target_ in the TV during IRE and (2) an electrical part through which IRE was applied to the TV (Figure 3). The container was designed using the open-source parametric 3D computer-aided design modeler FreeCAD V0.16 and 3D printed using polylactic acid (PLA) fiber. Furthermore, the water bolus wall consists of 0.2 mm polyvinyl chloride (PVC) film type claris (Walvis Products, Franeker, The Netherlands). The bolus was glued to the side of the container in which an electroporation cuvette was positioned. For the application of IRE, two copper-film tapes with a width of 10 mm were soldered to electrical wires that were connected to female banana connecters. The copper tapes were attached to the water bolus.

### 2.3. Temperature Measurements

Temperatures were measured using a single fiber optical temperature probe in the cell suspension (the TV) with a sample frequency of 1 Hz. The temperature probe is located in between the electrodes in the middle and at the bottom of the cuvette. Furthermore, the fiber optical temperature setup consisted of a rack-mounted Lumiterm X5 OEM temperature board connected to Lumiterm X5-True fiber optical temperature probes with a diameter of 1 mm (IPITEK, Carlsbad, CA, USA). The temperature probes have a resolution of 0.05 °C with an accuracy of 0.2 °C and are not affected by the presence of an electric field. Around the fiber optical temperature probe, a slight distortion can occur in the electric-field distribution. However, since the diameter of the optical probe (1 mm) is relatively small with respect to the width of the cuvette (10 mm), the electric-field distribution can be assumed to be homogeneously distributed in the remaining effective width (9 mm) of the cell suspension. Nevertheless, for future applications, it would be possible to remove the optical temperature probe just before the application of IRE and instantaneously reinsert it at the end of the treatment. This would avoid any distortion of the electric field-distribution. Please note that the main objective of this study was to present and validate an experimental set-up capable of maintaining stable temperatures “during” preclinical IRE experiments, and therefore, we chose to keep the optical temperature probe inside the target volume for the specific purpose of demonstrating the proof of principle. This does imply that, in the actual experimental use, there is no need for having a temperature probe inside the cuvette during the actual electroporation itself and that the optical temperature probe can be removed just before the application of IRE and instantaneously reinserted at the end of the treatment.

### 2.4. Cell Line and Cell Culture

In view of the relevance of the clinical application of IRE in pancreatic cancers [29,30], a pancreatic carcinoma cell line was selected for the validation of HyCHEED. The human pancreatic carcinoma Mia PaCa-2 (ATCC, Manassas, VA, USA) was grown in high glucose in Dulbecco’s Modified Eagle Medium (Life Technologies, Carlsbad, CA, USA) that was supplemented with 10% heat-inactivated fetal bovine serum (Gibco, Rockville, MD, USA) and 2 mmol·L^−1^ glutamine (Gibco, Rockville, MD, USA). The cells were cultured in a 37 °C incubator with humidified air supplemented with 5% CO_2_. After reaching a confluence of 90%, the cells were harvested via trypsinization (5 time diluted 0.5% Trypsin-EDTA, Gibco, Rockville, MD, USA) and suspended in the culture medium at a concentration of 0.98 × 10^6^–1.40 × 10^6^ cells·mL^−1^. The cell concentrations were determined using Trypan blue stain assay and an automated bright-field cell counter (LUNA^TM^ Automated Cell Counter, Aligned Genetics, Inc., Anyang-si, Korea). The protocol to determine the cell concentrations is described in Section 2.6.2. Please note that for the experiments in this study the culture medium was used in the cell suspension during the treatments. In Section 2.6 methods are discussed for the evaluation of the cells treated by HyCHEED.

### 2.5. Validation of HyCHEED

#### 2.5.1. Choice and Evaluation of Pulse Settings and Target Temperatures

The main aim of this article is to validate HyCHEED and to show its capability to operate with stable temperatures at different temperature levels. Therefore, without loss of generality two temperature values 37 °C and 46 °C were selected as T_target_, which represent the normothermic physiological (37 °C) and hyperthermic temperature (46 °C) that can develop during an IRE-treatment [20]. The reasons for choosing 46 °C are:

- a significant temperature difference compared to 37 °C, allowing us to demonstrate the capability of HyCHEED to operate at different temperatures;
- the likelihood of occurrence of 46 °C during typical IRE treatment [9,10,11];
- the limited direct cell death after brief exposure (~2–3 min) to 46 °C to allow the determination of the influence of the combined effects of IPE and temperature on both cell viability and specific conductivity [3,20,21].

At these temperatures, clinical IRE setting was applied to the TV consisting of a series of 90 pulses with a pulse duration of 90 μs and an electric-field strength (E [V·cm^−1^]) of 1250 V·cm^−1^ [11,29,31]. To examine the influence of the T_target_ on the TV during IRE, additional IRE setting was selected in which the E value was reduced to 500 V·cm^−1^ while maintaining the pulse duration and number.

Furthermore, the electric current and voltage of the first and the 90th pulses were evaluated to determine the capability of HyCHEED of producing typical electroporation pulses. The stability of the temperatures in the TV was assessed by calculating the mean and the standard deviation (SD) of the TV temperatures over the treatment time.

#### 2.5.2. Operation Protocol of HyCHEED

**Figure 4:**
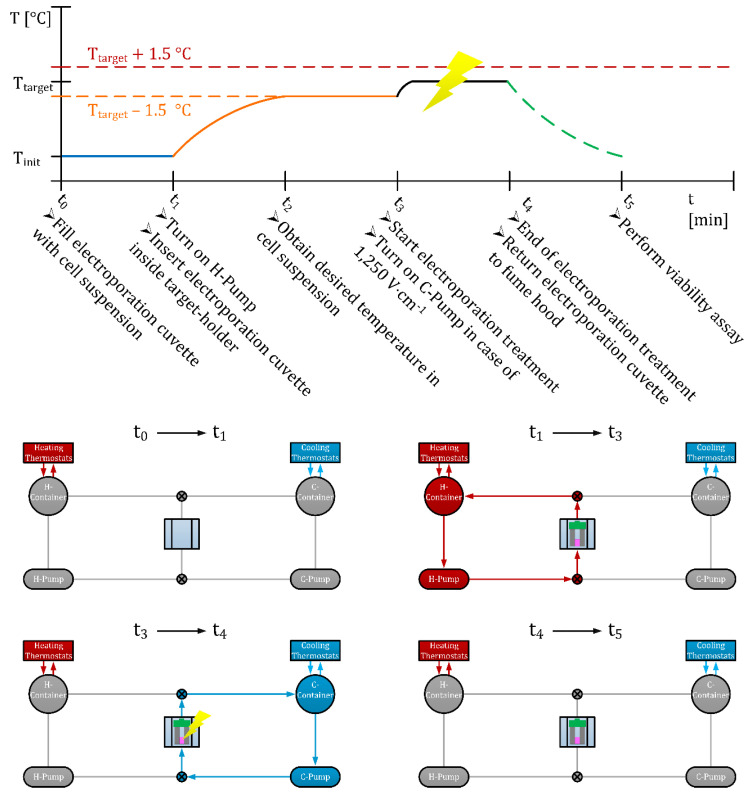
Time scheme of the application of HyCHEED in vitro. Please note that the red and orange dotted lines represent the temperature fluctuations around T_target_. Also note that at t_3_ H-Pump was switched to C-Pump when 1250 V·cm^−1^ was used.

This section describes the operation protocol of HyCHEED in vitro, as illustrated in Figure 4. Depending on the pre-specified T_target_ and electroporation settings, the demi-water in the containers was heated to temperatures specified in Table 1 (t_0_ → t_1_ in Figure 4). After obtaining the desired temperatures in the containers, ~300 μL of cell suspension was pipetted in the cuvette. To repeat, the culture medium was used in the cell suspension. Next, the fiber optical temperature probe was disinfected and inserted in the cell suspension in the cuvette inside the fume hood. Subsequently, the cuvette was slid along the water bolus, and copper tapes from above and the H-pump was turned on (t_1_ → t_2_ in Figure 4). After obtaining T [°C] ≥ T_target_—1.5 °C in the cell suspension (t_2_ → t_3_ in Figure 4), electroporation treatment was performed (t_3_ → t_4_ in Figure 4). The reason for starting at T_target_—1.5 °C was to suppress the expected temperature increase caused in the TV by the first series of pulses; especially in the case of 1250 V·cm^−1^. In case of control, the cuvette remained in the bolus for 90 s without electroporation during the period t_3_ → t_4_ in Figure 4. For the electric-field strength 1250 V·cm^−1^, the H-pump had to be switched to the C-pump for the temperature to remain within the accuracy of ±1.5 °C with respect to T_target_ (t_3_ → t_4_ in Figure 4). We chose ±1.5 °C as the maximal fluctuation range, since we noticed that during the IRE-treatment the temperature fluctuated between T_target_ and T_target_ ± 1.5 °C for 1250 V·cm^−1^. For 500 V·cm^−1^, the H-pump was only used since the temperature stability remained within ±1.5 °C. At the end of the treatment, the pumps were turned off, and the cuvette was returned into the fume hood (t_4_ → t_5_ in Figure 4).

### 2.6. Evaluation of In Vitro Experiments Using HyCHEED

HyCHEED can be used to perform in vitro IRE experiments at a pre-defined temperature level, as illustrated next. The evaluation of the in vitro experiments can be done by performing the cell viability assays in which a reagent is added to the cell suspension to quantitatively measure the proliferation of the living cells. Also, a typical characteristic of electroporation can be used as the evaluation process, namely the change in electrical specific conductivity (σ [S·m^−1^]). Previously, it was noted that the pore formation of the cells during IRE results in the increase of σ in the tissue [4]. Specifically, the change in the σ depends on the pulse settings (e.g., E and pulse number) and the temperature of the tissue [20]. To evaluate the in vitro experiments, the σ will be established during treatment of the first and the last pulse (90th), and directly after the treatment. For each assessment, n = 3 experiments were performed. More details are provided in the subsequent paragraphs.

#### 2.6.1. Establishing the Electrical Specific Conductivity of the Target Volume

To evaluate the in vitro experiments, the average electrical specific conductivity (σ_av_ [S·m^−1^]) will be used to determine the influence of temperature levels and electric-field strength on the pulse number. This will be done by calculating the ratio of the σ_av_ between the 90th and the first pulse. In addition, we will determine the influence of temperature and electric-field strength effects on the post-treatment σ_av_, which is the σ_av_ that is determined in ~30 s after treatment. Specifically, we will calculate the ratio between the post-treatment σ_av_ of the treatments with respect to the post-treatment σ_av_ of the control at 37 °C. Finally, to determine the influence of the presence of the electric field on σ_av_, we will compare the σ_av_ values of the 90th pulse with the post-treatment σ_av_.

**Figure 5:**
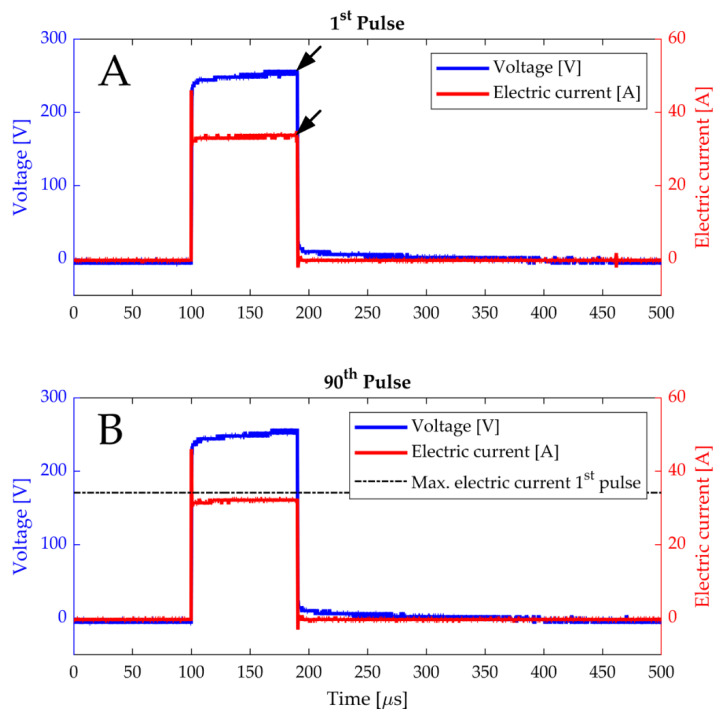
The electric current and voltage for 1250 V·cm^−1^ of (**A**) the first and (**B**) the 90th (last) pulse number as a function of time. In (**B**), a horizontal line was added to show the maximum of the electric current at the end of the first pulse. Also note that the black arrows in (**A**) represent the values of the electric current and voltage used in the calculation of the average electrical specific conductivity (σ_av_ [S·m^−1^]).

The average electrical specific conductivity of the cell suspension was determined using Ohm’s law,

(1) $$\frac{\Phi }{I}=\frac{1}{\sigma_{\mathrm{av}}}\cdot \frac{d}{A}$$

where Φ [V] is the measured voltage, I [A] is the measured electric current, d [m] is the distance between the electrode plates, and A [m^2^] is the surface of the TV that is contacting the electrode plate. To determine the change in the σ_av_ of the 90th pulse with respect to the first pulse, the σ_av_ values were calculated considering the extremities at the right side of the electric current and voltage of both the first and the 90th pulses (see the arrows representing the extremities at the right side in Figure 5A). Please note that, for the calculation of the σ_av_ values, the surface A was calculated by considering the initial height of the TV for the first pulse before its placement in the water bolus. For the 90th pulse, the height of the TV was considered after the removal of the cuvette from the bolus.

Around 30 s after the end of the control/treatment, before the removal of the electroporation cuvette from the bolus, a ~10 V pulse was applied to the TV. This 10 V is equivalent to 50 V·cm^−1^, which is below reversible electroporation threshold [32]. For the calculation of the post-treatment σ_av_ value, the surface A was calculated considering the height of the TV after the removal of the cuvette from the bolus.

#### 2.6.2. Cell Viability Assays/Reagents

To determine the cell viability after the controls/IRE-treatments, cell viability was assessed using PrestoBlue^TM^ cell viability assay and Trypan blue stain assay. The PrestoBlue^TM^ cell viability assay is a non-toxic resazurin-based method that functions as a cell viability indicator. Specifically, the metabolically active cells reduce the non-fluorescent blue dye (resazurin) into a highly fluorescent pink dye (resorufin) that can be quantitatively evaluated using fluorescence or absorbance measurements [33]. The Trypan blue stain assay is based on the Trypan blue diazo dye that is characterized by a molecular weight of 960 DA [34,35]. This molecule is a cell membrane-impermeable that can only enter the cytoplasm of the cell in case of compromised cell membrane, e.g., in case of reversible/irreversible electroporation and cell death. This assay could also be used to determine the cell concentration of the cell suspension as was done in this study.

After returning the electroporation cuvette to the fume hood post-treatment, the cell suspension was resuspended in the cuvette. Next, a total of 20.0 μL cell suspension was obtained and mixed with 20.0 μL Trypan Blue (Sigma-Aldrich, Saint Louis, MO, USA). Afterwards, 11.8 μL was inserted inside a cell counting slide (LUNA^TM^ Cell Counting Slides, Aligned Genetics, Inc., Anyang-si, South Korea) and the immediate cell viability and cell concentration were determined using the automated bright-field cell counter (LUNA^TM^ Automated Cell Counter, Aligned Genetics, Inc., Anyang-si, Korea) with a lens magnification of 1.47. The Trypan blue stain assay was performed ~8 min after the controls/IRE-treatments. Please note that the same protocol was used to determine the cell concentration of the cell suspension. Also note that this assay might provide an underestimation of the cell viability if applied within 10 min after treatment, due to the possible staining of viable reversibly electroporated cells. In addition, a number of cells within the TV could need more time to relapse or to recover, which could further reduce the accuracy of this assay. Therefore, in this study the cell viability was mainly determined using PrestoBlue^TM^ cell viability assay, to provide more time (~2 days) for cell recovery or cell relapse. The Trypan blue stain assay was only used to present the cell morphology and viability before and after IRE-treatment.

For the PrestoBlue^TM^ cell viability assay, 6.3 μL of the treated cell suspension was plated in a 96 well plate that was returned to the incubator at the end of the experiment. After ~2 days, the old DMEM was removed and a 100.0 μL fresh DMEM including a 10 times diluted Molecular Probes^TM^ PrestoBlue^TM^ Cell Viability Reagent (Thermo Fisher Scientific, Waltham, MA, USA) was added to the wells that contain cells. Subsequently, the well plate was returned to the incubator. After ~5 h, the change in the fluorescence of the PrestoBlue reagent due to the presence of viable cells was measured using Synergy 2 Multi-Mode Microplate Reader (BioTek, Agilent Technologies, Winooski, VT, USA) with the excitation/emission wavelengths set at 560/590 nm.

#### 2.6.3. Statistical Analyses

In this study the data are presented as mean ± standard deviation (± SD) of at least three independent experiments. Furthermore, a two-tailed Student’s *t*-test (two groups; unequal variance) was utilized to determine the significant difference between temperatures for every experiment. The criterion for statistical significance was set to be p < 0.05. The statistical analyses were performed using Excel 2016 (Microsoft Office, Microsoft, Redmond, WA, USA).

## 3. Results

### 3.1. Validation of HyCHEED

#### 3.1.1. Read-Outs of Electrical Parameters

To demonstrate the capability of HyCHEED in producing typical clinical electroporation pulses, examples of the electric current and voltage readouts of the first and the 90th pulse are presented in Figure 5 for 1250 V·cm^−1^ at 46 °C. Here, the maximal electric current and voltage were 34.1 A and ~254 V during the first pulse, and 32.5 A and ~254 V during the 90th pulse. At the 90th pulse, the electric current was slightly reduced due to the reduction of the height of the target volume (TV) caused by gas formation as a result of water electrohydrolysis [36]. Specifically, for 1250 V·cm^−1^ the initial height of the TV (~15 mm) decreased with ~1 mm (~7%) at 37 °C and ~2 mm (~13%) at 46 °C, while the height for 500 V·cm^−1^ decreased far less than 1 mm at 37 °C and 46 °C. Despite the increase in the electrical specific conductivity (σ [S·m^−1^]) of the TV during the IRE treatment in case of Figure 5, the trade-off between the increase in the σ and reduction in the height of the TV resulted in the slightly decrease of the electric current in the last pulse with respect to the first pulse.

Furthermore, the shapes of the electric current and voltage resemble the shapes of typical electroporation pulses shown in Figure 8 of [37]. Specifically, the peak at the start of the electric current is generated due to the movements of charged ions and molecules, and the reorientation of the electric dipoles, after which the electric current slightly increases due to the pore formations in the cell membranes [38].

#### 3.1.2. Temperature Measurements

**Figure 6:**
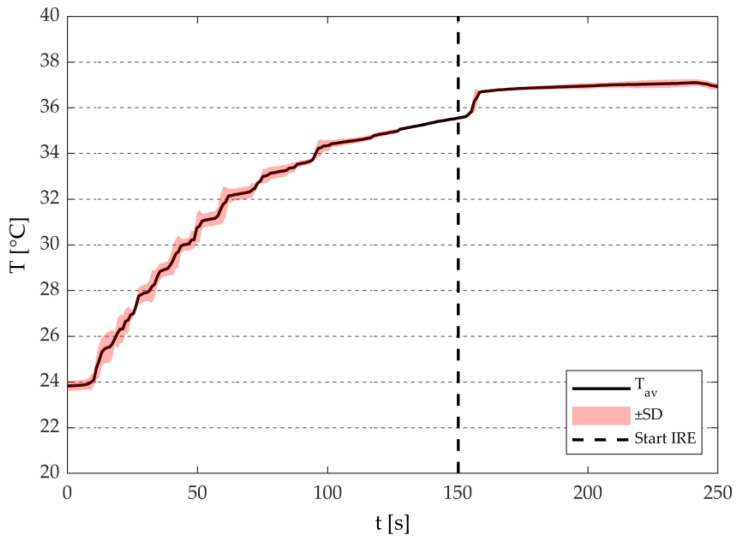
An illustration of the average temperature evolution over time (T_av_; n = 3) during the treatment at 37 °C for 1250 V·cm^−1^. The solid line represents the average temperature, pink area represents the standard deviation (±SD), and the dashed line represents the start of the IRE treatment.

Figure 6 illustrates the average temperature evolution over time in the electroporation cuvette during the IRE-treatment. Here, the temperature evolution was provided as an example and omitted in the rest of the experimental results. The time between the insertion of the cuvette in the T-holder and the start of the treatment is ~2.3 min. The mean temperatures over the treatment time are shown in Table 2 demonstrate that T_target_ could be obtained within ±1.5 °C with respect to desired temperature, independent of the selection of T_target_, or the pulse settings.

### 3.2. Effects of Thermally Controlled Irreversible Permeabilization on the Electrical Specific Conductivity and Cell Viability

#### 3.2.1. Electrical Specific Conductivity

**Figure 7:**
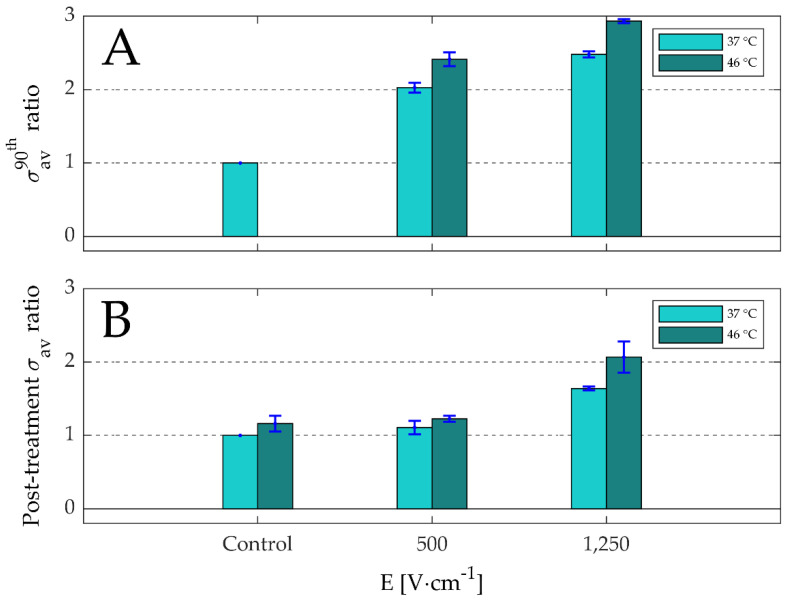
The ratios of the average electrical conductivities (σ_av_ [S·m^−1^]) of the 90th pulse (**A**) and post-treatment (**B**) with respect to the post-treatment σ_av_ of the control at 37 °C (0.75 S·m^−1^) are shown as a function of the electric-field strength (E [V·cm^−1^]) and target temperature (n = 3; including ±SD). The data are provided in Supplementary Materials. Note that the post-treatment σ_av_ was measured at about 30 s after the 90th pulse.

Figure 7 shows the σ_av_ ratios of the 90th pulse (Figure 7A) and the post-treatment (Figure 7B) with respect to the post-treatment σ_av_ of the control at 37 °C as a function of electric-field strength and target temperature. The data, including the σ_av_ of the culture medium excluding cells, are provided in Supplementary Materials. These results illustrate increase of σ_av_ ratio with respect to the control at 37 °C as a function of the following effects:

1. Temperature only:
  - In Figure 7B, the σ_av_ ratio for the control at 46 °C increased by 1.2-fold with respect to the control at 37 °C.
2. Electric-field strength only; during the pulse in the presence of the electric field (focus on Figure 7A):
  - In Figure 7A, the σ_av_ ratios for 1250 V·cm^−1^ at 37 °C and 46 °C increased by 2.5 and 2.9-folds, while the σ_av_ ratios for 500 V·cm^−1^ at 37 °C and 46 °C increased by 2.0 and 2.4-folds.
  - In Figure 7B the σ_av_ ratio for the control at 46 °C increased by 1.2-fold, while in Figure 7A, the σ_av_ ratios for 500 V·cm^−1^ and 1250 V·cm^−1^ at 46 °C increased by 2.4 and 2.9-fold.
3. Electric-field strength only; post-treatment in the absence of the electric field (focus on Figure 7B):
  - In Figure 7B, the σ_av_ ratios for 1250 V·cm^−1^ at 37 °C and 46 °C increased by 1.2 and 2.1-folds, while the σ_av_ ratios for 500 V·cm^−1^ at 37 °C and 46 °C increased by 1.1 and 1.6-folds.
  - In Figure 7B, the σ_av_ ratio for the control at 46 °C increased by 1.2-fold, while in the σ_av_ ratios for 500 V·cm^−1^ and 1250 V·cm^−1^ at 46 °C increased by 1.6 and 2.1-fold.
4. Presence of electric field during the treatment:
  - In Figure 7A, the σ_av_ ratios for 500 V·cm^−1^ and 1250 V·cm^−1^ during the 90th pulse at 37 °C increased by 2.0 and 2.5-folds, while in Figure 7B the σ_av_ ratio post-treatment increased by 1.11 and 1.64-folds for the same treatment parameters.
  - In Figure 7A, the σ_av_ ratios for 500 V·cm^−1^ and 1250 V·cm^−1^ during the 90th pulse at 46 °C increased by 2.4 and 2.9-folds, while in Figure 7B the σ_av_ ratio post-treatment increased by 1.2 and 2.1-folds for the same treatment parameters.
5. Combined effects of electric-field strength and temperature; during the 90th pulse in the presence of the electric field (focus on Figure 7A):
  - In Figure 7A, the σ_av_ ratios for 500 V·cm^−1^ and 1250 V·cm^−1^ during the 90th pulse at 46 °C increased by 2.4 and 2.9-folds, while the σ_av_ ratios for the same pulse parameters at 37 °C increased by 2.0 and 2.5-folds.
6. Combined effects of electric-field strength and temperature; post-treatment in the absence of the electric field (focus on Figure 7B):
  - In Figure 7B, the σ_av_ ratios for 500 V·cm^−1^ and 1250 V·cm^−1^ post-treatment at 46 °C increased by 1.2 and 2.1-folds, while the σ_av_ ratios for the same pulse parameters at 37 °C increased by 1.1 and 1.6-folds.

**Figure 8:**
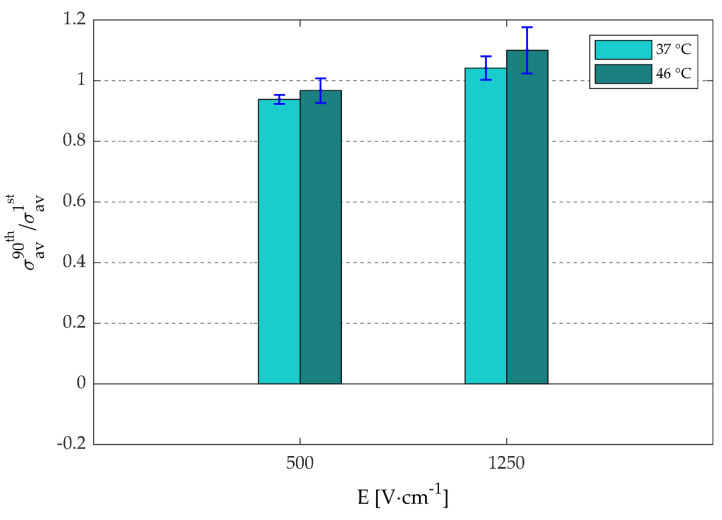
The average ratio (n = 3; including ±SD) between the average electric conductivities (σ_av_ [S·m^−1^]) of the 90th and the first pulse are shown as a function the electric-field strength (E [V·cm^−1^]) and target temperatures. The data are provided in Supplementary Materials Table S2.

In Figure 8, the ratios between the σ_av_ of the 90^th^ pulse and the first pulse are presented as a function of electric-field strength and target temperature to determine the influence of the pulse number. The data used in this figure are provided in Supplementary Materials Table S2. These results reveal that for 500 V·cm^−1^, the σ_av_ of the 90th pulse is slightly lower than the σ_av_ of the first pulse, with an average ratio of ~0.95, while for 1250 V·cm^−1^, the σ_av_ of the 90th pulse is slightly higher than the σ_av_ of the first pulse, with an average ratio of ~1.05, implying that σ_av_ hardly increase as function of pulse number. Furthermore, Figure 8 also demonstrates that increase in temperature results in the increase of the σ_av_ ratios, implying that higher temperatures would increase the electrical specific conductivity of the target volume during IRE.

#### 3.2.2. Cell Viability

**Figure 9:**
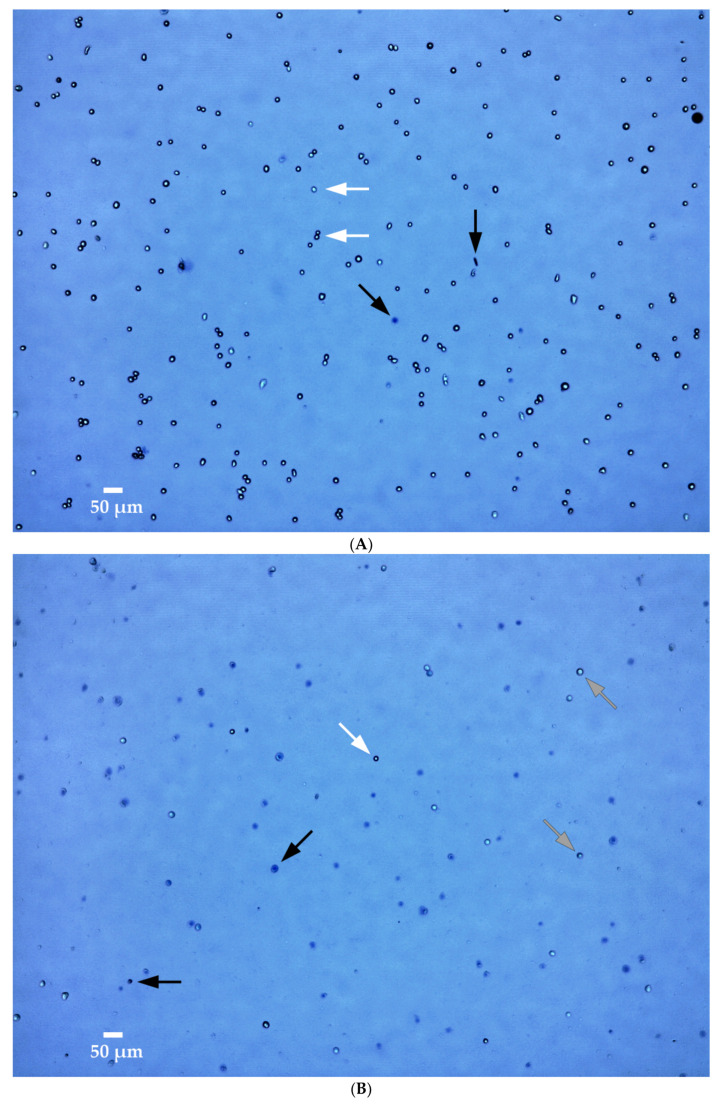
Example of cell viability assay (Trypan blue assay) performed on the cell suspension maintained at 37 °C (**A**) without the application of irreversible electroporation, and (**B**) after application of irreversible electroporation (electric field strength of 1250 V·cm^−1^). The length of the scale bar in both images represents 50 μm. Vital cells are noted by light blue circles with dark edges (indicated by white arrows), while electroporated cells are noted by blue circles with less dark edges (indicated by gray arrows) and dead or dying cells are noted by dark blue, or dim circles (indicated by black arrows). Note that the cell viability was determined using an automated bright-field cell counter (LUNA^TM^ Automated Cell Counter, Aligned Genetics, Anyang-si, Inc., South Korea) with a lens magnification of 1.47.

Figure 9 shows examples of the viability assay of untreated and treated cell suspensions at 37 °C. Specifically, the cell suspension in Figure 9A was an untreated control and had a cell concentration of 1.17·10^6^ cells·mL^−1^, while the cell suspension in Figure 9B was exposed to 1250 V·cm^−1^ and had a much reduced cell concentration of 0.04·10^6^ cells·mL^−1^ (i.e., 97% cell death). Since HyCHEED is capable of performing irreversible electroporation treatment while maintaining a stable predefined temperature, these results show that irreversible permeabilization at 1250 V·cm^−1^ is sufficient to induce cell ablation with limited addition of heat.

**Figure 10:**
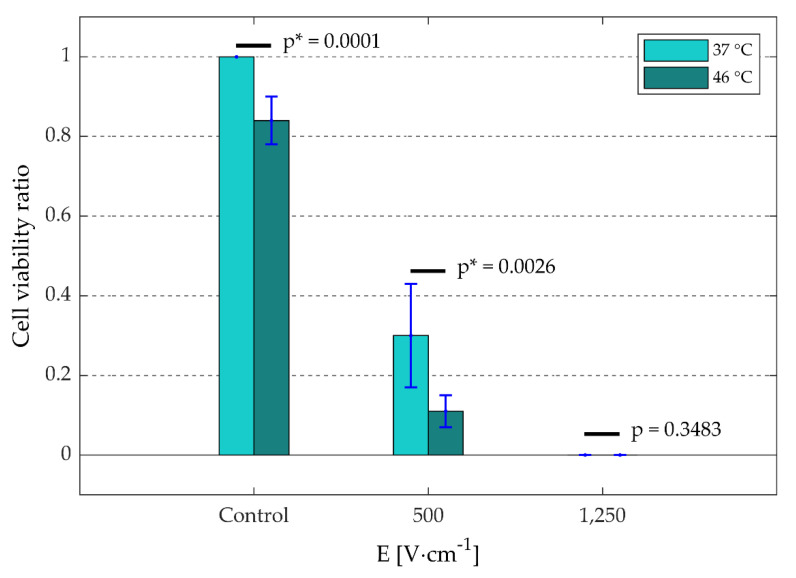
The cell viability ratio (n = 3; including ± SD) of cell suspensions as a function of electric-field strength (E [V·cm^−1^]) and target temperature with respect to the control at 37 °C. For both control and 500 V·cm^−1^, the cell viability ratio at 46 °C significantly differ from the ratio at 37 °C (*p* ≤ 0.0026; * indicates significant difference). Please note that during the control/treatments, the culture medium was used in the cell suspension.

In Figure 10, the viability ratio of the cell suspensions are presented as a function of electric-field strength and target temperature. As in Figure 7, this figure also shows cell death as a function of (1) only temperature (control, 46 °C), (2) only irreversible permeabilization effect (1250 V·cm^−1^, 37 °C), and (3) combined effects of irreversible permeabilization and temperature (500 V·cm^−1^, 46 °C). Specifically, it shows that the exposure of untreated cell suspension to 46 °C for ~2–3 min results in 15% of thermal ablation. By treating the cells with 500 V·cm^−1^, a total cell death of 70% was obtained at 37 °C, which increases to 90% at 46 °C due to an additional combined thermal-IPE ablation. For the treatment of the cells with 1250 V·cm^−1^, a total ablation of 100% was obtained for both 37 °C and 46 °C. These results show that, at relatively low electric-field strengths, temperature not only plays a significant role in the increase of the electrical specific conductivity of the target volume, but also in the increase of the cell death in the target tissue.

## 4. Discussion

### 4.1. Main Research Objective: HyCHEED Was Validated

This study demonstrated that the hydraulically controllable heat exchange electroporation device (HyCHEED) is capable of applying steady electrical pulses to the target volume (Figure 5) while maintaining stable temperatures during the electroporation treatment for clinically relevant temperatures and electroporation settings. This was done while limiting temperature fluctuations to ±1.5 °C, as was shown in Figure 6 and Table 2. In addition, the proper performance of HyCHEED was evident from the dependence of the cell viability in Figure 9 and Figure 10 on target temperature and electric-field strength, corresponding to the data from the previous studies [27,28]. Specifically, increase of target temperatures and electric field results in increase of cell death.

### 4.2. Secondary Research Objective: Irreversible Electroporation Characteristics Are Influenced by Electric-Field Strength and Temperature

In this study we showed that the increase of cell death (Figure 10) and electrical specific conductivity (Figure 7) due to IRE depends on electric-field strength, temperature, and the combined effects of both parameters. These findings correspond with the conclusion in our previous study [20], in which we reported the presence of these effects in the IRE-treated regions. In our previous study, we performed a systematic review using various numerical models from the literature to assess the degree of mild-hyperthermic and thermally ablative effects caused by IRE within IRE-treated region. We concluded that while 5% of the IRE-treated region was thermally ablated, in 30% of the treated region, a combined effect of both elevated temperature and irreversible permeabilization occurs. The remaining 65% ablation was only caused by irreversible permeabilization. The results in this study signify the feasibility of the presence of these effects during a typical clinical IRE treatment, depending on the spatially-temporally depended magnitudes of the electric-field strength and the temperature increase.

Moreover, Figure 7 also showed the dependence of the electrical specific conductivity post-treatment on the electric-field strength, temperature, and combined effects of both parameters, even after the electric field was switched off after treatment. In addition, it shows that the electrical specific conductivity decreases after treatment, which could be attributed to membrane resealing at the end of the treatment, as was shown in Ivorra et al. [39,40]. Ivorra et al. investigated the electrical specific conductivity within, in between, and after (reversible and irreversible) electroporation pulses in healthy and cancerous tissue in rodents. Similarly, they found a rapid drop in the electrical specific conductivity after electroporation.

Furthermore, Figure 8 shows that the electrical specific conductivity ratios between the 90th and the first pulses are almost equal to 1 for 500 V·cm^−1^ and 1250 V·cm^−1^. Accordingly, the combined results of both Figure 7A and Figure 8 suggests that the electrical specific conductivity mainly depends on the electric field and temperature, and less on the pulse number. These results are in accordance with the previous studies, which also showed that the electrical specific conductivity ratios between the last and the first pulses are ~1 in case of eight pulses [39,41]. It must be noticed that, in Figure 8, the average ratio is slightly below 1 for 500 V·cm^−1^, while it is slightly above 1 for 1250 V·cm^−1^. This can be explained by the combined effects of electrical specific conductivity increase and height reduction of the target volume caused by gas formation as a result of water electrohydrolysis. E.g., despite the decrease of the height of the target volume for 1250 V·cm^−1^ in the order of 1.5 mm, an almost complete ablation was performed, with as a result a slight increase in the σ_av_ of the 90th with respect to the first pulse.

Furthermore, it was also stated in the literature that the electric-field strength and temperature level play a critical role during the IRE treatment [8,20,27]. Specifically, a larger electric-field strength (for E > E_IRE(th)_) results in a larger ablation area [1,8,27], and temperature levels above a certain threshold would result in instantaneous thermal damage [8,9]. These facts were also observed in Figure 10, in which the exposure to 46 °C resulted in thermal ablation of 15% of the cell suspension, while the increase of the electric-field strength from 500 V·cm^−1^ to 1250 V·cm^−1^ at 37 °C resulted in an IPE ablation increase from 70% to 100%. In addition, Figure 10 illustrated at 500 V·cm^−1^ that thermal elevation enhances the IPE ablation with additional 20% more cell death as a result. These data correspond with the data of Edelblute et al., which also showed that the enhancement of ablation increases with increasing temperature [27].

### 4.3. HyCHEED Is Suitable for Investigation of Electroporation Treatment Modalities

HyCHEED is suitable for investigating specific user-selected temperature levels on permeabilization during electroporation, such as in reversible, irreversible, and high-frequency irreversible electroporation (HF-IRE). This, to assess in more detail the possible contribution of thermal effects to the permeabilization effect (e.g., determine influence of elevated temperature levels on E_IRE(th)_). Here, the temperature levels could be other than the 37 °C and 46 °C used in this paper. Due to the high-frequency electric current of HF-IRE, the demi-water can become lossy, thus lowering its impedance [42,43]. As a consequence, part of the high-frequency electric current would flow through the demi-water, possibly resulting in deterioration of the treatment efficacy. Still, for this technique, commercially available low viscous silicone oil can be used instead of demi-water to maintain the treatment efficacy, due to its lossless properties.

For the electroporation modalities, HyCHEED provides a temperature-controlled environment, in which the direct effect of a reasonably homogeneously distributed electric-field strength on electroporation can be determined. This can be done on several tissue types at user-defined temperature levels with limited temperature fluctuations. By determining the electrical properties as a function of electric-field strength and temperature of, e.g., human ex vivo tissues, we could apply this knowledge to improve the modeling of the electrical properties as used in numerical treatment planning for preparation, evaluation and optimization of the electroporation treatments [44,45]. In the numerical treatment planning of electroporation treatments, numerical calculations are performed on realistic 3D tumor models constructed from patient-specific medical images before the treatment. Here, the parameters of pulses and electrodes, such as pulse voltage, electrode number, and configuration, are numerically optimized for each patient.

### 4.4. HyCHEED Is Suitable for Investigation of Radio-Frequency Treatments

Radio-frequency (RF) treatments are treatment modalities in which a target tissue is exposed to high-frequency electromagnetic waves with the aim to induce thermal elevation within the target tissue [3]. In case of mild temperature elevations (T ≤ 45 °C), the RF treatments can be used to enhance the therapeutic effects of adjuvant treatment modalities, such as radiotherapy and chemotherapy [46,47]. For large temperature elevation (T ≥ 60 °C), the RF treatments are used as thermal ablation modalities, such as in radio-frequency ablation [48]. Despite the purpose of inducing temperature elevations, several studies suggested that the non-thermal RF waves also affect the target tissue next to hyperthermia [49,50,51]. For instance, Ware et al. investigated the influence of RF waves on the physical parameters of the pancreatic cell lines in the range of 33–39 °C and found changes in cell topography, morphology, motility, adhesion, and division as the result of exposure to RF waves [49]. Another example is a study performed by Wust et al. [51], in which preclinical treatments in human colon cancer cell lines were performed by comparing water bath heating with RF hyperthermia at 42 °C. The authors found that RF hyperthermia significantly reduced proliferation and clonogenicity compared to water bath hyperthermia, suggesting additional effects of non-thermal RF. In such studies, HyCHEED can be used to investigate the influence of RF waves on cell suspensions for several temperature levels within a well-controlled small temperature range. As mentioned above, the demi-water can be replaced by commercially available low viscous silicone oil that is lossless to avoid its impedance of becoming lossy due to the high-frequency electric current induced by the RF treatments.

### 4.5. Future Directions

HyCHEED offers the opportunity to determine the influence and the contribution of various temperature levels on several bio-electromagnetic techniques and electromagnetic properties (e.g., electrical specific conductivity). However, it is important to notice that the materials are temperature dependent and, therefore, it is important to choose materials with appropriate service temperatures to avoid setup failure. We used materials suitable for the present temperature range of 37–46 °C. Furthermore, by designing custom plate electrodes, HyCHEED would also be suitable for the use of ex vivo or 3D agarose targets. The present HyCHEED system was designed for performing temperature-controlled IRE for a single cuvette, the hydraulic circuit could be redesigned to allow application of temperature-controlled IRE in multiple cuvettes in parallel, which can be helpful for accelerating research when testing different cell lines as often required in today’s journal standards.

## 5. Conclusions

In this study, we presented a hydraulically controllable heat exchange electroporation device (HyCHEED) that is capable of successfully treating the target volume with various electroporation settings at stable pre-defined clinically relevant temperature levels. This provides an excellent basis for important preclinical experiments to assess the extent of the contribution of thermal effects to the IRE ablation zone and determine the therapeutic ratio between thermal damage and irreversible permeabilization effects.

## Figures and Tables

**Table 1:** Average temperatures of the demineralized water in the heating (H) and cooling (C) containers. These temperatures depend on the target temperature (T_target_ [°C]) and IRE settings. The temperatures of H-containers were used for both 500 V·cm^−1^ and 1250 V·cm^−1^, while temperatures of C-containers were only used for 1250 V·cm^−1^.

T_target_ [°C]	Temperature of H-Container [°C]	Temperature of C-Container [°C]
**37**	37.5	32.4
**46**	46.1	44.2

**Table 2:** The mean temperature over the treatment time (n = 3; including ±SD) acquired from the treatments as a function of target temperature (T_target_ [°C]) and electric-field strength (E [V·cm^−1^]).

T_target_ [°C]	E [V·cm^−1^]	
	500	1250
**37**	36.75 ± 0.58	36.86 ± 0.34
**46**	46.44 ± 0.82	46.66 ± 0.70

## Supplementary Materials

The following are available online at https://www.mdpi.com/1424-8220/20/21/6227/s1, Table S1: The post-treatment σ_av_ of the target volume including the ratios with respect to the post-treatment σ_av_ of the control at 37 °C, Table S2: A summary of σ_av_ obtained from the first and the 90th pulses including the ratios with respect to the σ_av_ of the first pulse.

## Abbreviations and Definitions

**Abbreviations and symbols of quantities**	**Unit**	**Definition**
C-container		Container with demineralized water intended to cool off a target volume.
Demi-water		Demineralized water.
IRE		Irreversible electroporation.
IPE		Irreversible permeabilization effect (we chose IPE instead of IRE effect to distinguish between only the permeabilization effect, and the permeabilization and the thermal effects jointly produced by IRE).
H-container		Container with demineralized water intended to heat a target volume.
HF-IRE		High-frequency irreversible electroporation.
HyCHEED		Hydraulically controllable heat exchange electroporation device.
NA		Not applicable.
Post-treatment σ_av_	[S·m^−1^]	Average electrical specific conductivity of a target volume that is measured after the treatment.
T-holder		Target holder.
TV		Target volume.
**E**	[V·m^−1^] or [V·cm^−1^]	Electric-field vector.
**J**	[A·m^−2^]	Electric-current density.
**J** · **E**	[W·m^−3^]	The Joule Heating term; the heat generation rate per unit volume.
c_p_	[J·kg^−1^·°C^−1^]	Specific heat capacity of a medium.
d	[m]	Distance between electrode plates of an electroporation cuvette.
f_P_	[Hz]	Pulse frequency.
H_V_	[W·m^−3^·°C^−1^]	Volumetric heat transfer coefficient between the target volume and its direct ambiance.
t	[s] or [min]	Time.
t_0_, t_1_, …, t_5_	[min]	Time points.
t_init_	[s]	Initial time at which irreversible electroporation treatment starts.
t_P_	[s]	Pulse duration.
σ	[S·m^−1^]	Electrical specific conductivity of a target volume.
σ_av_	[S·m^−1^]	Average electrical specific conductivity of a target volume.
A	[m^2^]	Surface of a target volume that is contacting the electrode plate of an electroporation cuvette.
E = |**E** |	[V·m^−1^] or [V·cm^−1^]	Magnitude of an electric-field vector.
E_IRE(th)_	[V·m^−1^]	Electric-field threshold of irreversible electroporation; minimum electric-field value that ablates target cells/tissue during the irreversible electroporation.
I	[A]	Measured electric current.
SD		Standard deviation.
T	[°C]	Temperature.
T_target_	[°C]	Target temperature.
T_A_	[°C]	Temperature of the direct ambient of the target volume.
T_av_	[°C]	Average temperature evolution over time.
Φ	[V]	Measured voltage.

## Appendix A—Mathematical Description of Stable Temperature Control in Treated Volume

The description of the thermal balance within a small target volume (TV; 300 μL of cell suspension) that is presented as a purple cuboid in Figure 1 is based on the heat transfer equation

(A1) $${\rho c}_{p}\frac{\partial T}{\partial t}=-H_{V}\cdot \left(T-T_{A}\right)+J\cdot E\cdot t_{P}\cdot f_{P}$$

where ρ [kg·m^-3^] is the mass density, c_p_ [J·kg^−1^·°C^−1^] is the specific heat capacity, T [°C] is the temperature, t [s] is the time, and **J** · **E**· t_P_ · f_P_ [W·m^-3^] is the scaled heat generation rate per unit volume (the joule-heating term; **J** · **E** [W·m^−3^]) over the period 1/f_P_ [s], with **J** [A·m^−2^] as the electric-current density, **E** [V·m^−1^] as the electric field, t_P_ [s] as the pulse duration, and f_P_ [Hz] as the pulse frequency [20]. The term H_V_ · (T − T_A_) represents the rate of heat flow into the TV and is utilized when, e.g., thermal advection is applied through water flow surrounding the TV. Here, H_V_ [W·m^-3^·°C^−1^] is the volumetric heat transfer coefficient between the TV and its direct ambiance with the temperature T_A_ [°C]. This term is used to heat the TV, as was shown in Figure 1A (the term is represented by orange arrows), or to cool it down, as in Figure 1C (the term is represented by blue arrows). Since we only apply IRE pulses in this study, the joule-heating term can have the form

(A2) $$J\cdot E=\sigma \cdot E^{2}$$

with σ [S·m^−1^] as the electrical specific conductivity of the TV, and **E** [V·m^−1^] as the magnitude of the electric field (other term is the electric-field strength) to which the TV is exposed [20]. An example of electric pulses are shown in Figure 1B with t_init_ [s] as the initial time at which the electric pulses are applied.

To maintain a stable temperature in the TV at the target temperature (T_target_ [°C]; desired temperature at which electroporation is applied), the temporal temperature change must satisfy

(A3) $$\frac{\partial T}{\partial t}=0$$

resulting in two distinct scenes:

1. Pretreatment (E = 0 V·m^−1^), HyCHEED has to heat the TV up to T_target_. This implies that at t = 0 s the temperature of the TV is lower than T_A_, and in time the temperature must reach T_target_. Therefore, T_A_ = T_target_. See Figure 1A.
2. During the treatment (E > 0 V·m^−1^), a stable temperature is maintained when the temperature rise caused purely by the electric pulses (Figure 1B) is combined with the temperature decrease caused solely by the cooling effect of term H_V_ · (T − T_A_) (Figure 1C) to obtain a steady temperature as in Figure 1D. To satisfy this condition, T_A_ must be adjusted at t = t_init_ such that T_A_ < T_target_. Using the combination of Equations (A1) and (A3), we can calculate T_A_ by reducing Equation (A1) into

(A4) $$\sigma \cdot E^{2}\cdot t_{P}\cdot f_{P}=H_{V}\cdot \left(T-T_{A}\right)$$

resulting in the steady state solution

(A5) $$T=T_{A}+\frac{\sigma \cdot E^{2}\cdot t_{P}\cdot f_{P}}{H_{V}}$$

or

(A6) $$T_{target}=T_{A}+\frac{\sigma \cdot E^{2}\cdot t_{P}\cdot f_{P}}{H_{V}}$$
