# Supplementary material for: HyCHEED System for Maintaining Stable Temperature Control during Preclinical Irreversible Electroporation Experiments at Clinically Relevant Temperature and Pulse Settings
Source: Sensors (Basel). 2020 Oct 31;20(21):6227. doi: 10.3390/s20216227 (PMC7662544; doi:10.3390/s20216227)
Supplement: Supplementary file 1 [file sensors-20-06227-s001.pdf]

sensors

MDPI

Supplementary Materials

Table S1. The average electrical specific conductivities of the target volume post-treatment (post-treatment σ_{av} [S·m^{-1}] ± SD; n = 3, red table cells), including the ratios with respect to the post-treatment σ_{av} of the control (0 V·m^{-1}) at 37 ˚C (blue table cells). The data are presented as a function of both electric-field strength (E [V·cm^{-1}]) and target temperature (T_{target} [˚C]).

			E		
	**T_{target}**	**Culture medium**	**0 V·cm^{-1} (Control)**	**500 V·cm^{-1}**	**1,250 V·cm^{-1}**
**Post-treatment σ_{av} [S·m^{-1}]**	**37 ˚C**	0.81 ± 0.05	0.75 ± 0.08	0.83 ± 0.07	1.23 ± 0.02
	**46 ˚C**	0.93 ± 0.07	0.87 ± 0.08	0.92 ± 0.03	1.55 ± 0.16
**Post-treatment ratio of σ_{av} between treated target volume and control at 37 ˚C**	**37 ˚C**	1.07 ± 0.06	1.00	1.11± 0.09	1.64 ± 0.03
	**46 ˚C**	1.24 ± 0.10	1.16 ± 0.11	1.23 ± 0.04	2.07 ± 0.21

Table S2. A summary of the data of the average electrical specific conductivities (σ_{av} [S·m^{-1}] ± SD; n = 3) obtained from the 1st and the 90th pulses, including the ratios with respect to the σ_{av} of the 1st pulse. The data are presented as a function of both electric-field strength (E [V·cm^{-1}]) and target temperature (T_{target} [˚C]). Here, NA is defined as Not Applicable.

			E	
	**T_{target}**	**Pulse number**	**500 V·cm^{-1}**	**1,250 V·cm^{-1}**
**σ_{av} [S·m^{-1}]**	**37 ˚C**	**1st**	1.62 ± 0.04	1.79 ± 0.09
		**90th**	1.52 ± 0.05	1.86 ± 0.03
	**46 ˚C**	**1st**	1.88 ± 0.01	2.01 ± 0.12
		**90th**	1.81 ± 0.07	2.20 ± 0.02
**Ratio between σ_{av} of 90th and 1st pulse numbers**	**37 ˚C**	**NA**	0.94 ± 0.02	1.04 ± 0.04
	**46 ˚C**	**NA**	0.97 ± 0.04	1.10 ± 0.08
